# Low magnitude of tensile stress represses the inflammatory response at intervertebral disc in rats

**DOI:** 10.1186/s13018-015-0159-y

**Published:** 2015-02-07

**Authors:** Chao Han, Xin-long Ma, Tao Wang, Jian-xiong Ma, Peng Tian, Jia-cheng Zang, Jing-bo Kong, Xiao-dan Li

**Affiliations:** Department of Orthopaedics, Tianjin Medical University General Hospital, No.154 Anshan Road, Heping District, Tianjin City, 300052 PR China; Tianjin Hospital, No. 406 Jiefang South Road, Hexi District, Tianjin City, 300211 PR China; Department of Anesthesiology, Tianjin First Central Hospital, No.24 Fukang Road, Nankai District, Tianjin City, 300192 PR China

**Keywords:** Mechanical stress, Tail suspension, Anti-inflammation, Interleukin-17, Interleukin-1β, Inducible nitric oxide synthase

## Abstract

**Objective:**

This study aims to determine if the involvement of tensile stress affects the expressions of inflammatory cytokines interleukin-17(IL-17), interleukin-1β (IL-1β), and inducible nitric oxide synthase (iNOS) at intervertebral discs *in vivo*.

**Material and method:**

Sixty-four female Sprague–Dawley rats were randomly divided into four groups: sham, tail-suspended (TS), tail-suspended with needle puncture (TSNP), and single-needle puncture (SNP) groups. A tail-suspension device provides low magnitude of tensile stress (2.45 Newton (N)), and aseptic needle puncture on the tail disc induces inflammatory response. After 4 weeks, the treated discs were harvested for histologic analysis, quantitative real-time reverse transcription-polymerase chain reaction (RT-qPCR), and enzyme-linked immunosorbent assay (ELISA).

**Result:**

Pathological examination demonstrated that compared to the sham group, the morphologies of nucleus pulposus (NP) and anulus fibrosus (AF) in TS, SNP, and TSNP groups displayed degenerative changes in varying degrees. Results from RT-qPCR showed that IL-17 and iNOS mRNA expression levels were significantly higher in both TSNP and SNP groups than those in the sham groups. Expression of IL-17 and iNOS are not significantly different between the sham and TS groups (*P >* 0.05). Compared with the SNP group, the mRNA expression of IL-17 and iNOS in the TSNP groups were markedly decreased (*P* < 0.05). The regulation of IL-1β and IL-17 detected by ELISA was coincident with the qRT-PCR results.

**Conclusion:**

The results from this study suggested that relatively low magnitude tensile stress might play an essential role in the anti-inflammatory process and the relief of low-back pain (LBP).

## Introduction

Motion-based therapy is commonly used in clinical practice for the treatment of low-back pain (LBP), and many clinical evidences have convinced its effectiveness, especially for those with lumbar intervertebral disc herniation [1,2]. However, the clinical effect and mechanism of tensile force for LBP are still unclear [3,4]. Due to the ethics and existence of other elements adjacent to the spine, the rodent-tail model, on account of the similarities in the biologic and biomechanical properties to human lumbar disc [5], has become an excellent model to investigate the biochemical responses of the disc cells to mechanical force [6]. Current researches on the tensile stress focus more on the studies *in vitro*. Some tissues responding to mechanical stress have showed positive effects with enhanced cellular proliferation, matrix production, and relative gene expression [7-9].

Tensile stress does not work on the disc cell alone. In fact, increased evidence showed that inflammatory mediators may play an essential role in the regulation of the LBP in the intervertebral disc herniation and the mechanical force may combine with the inflammatory reaction to contribute the processing of disc diseases [10]. In the past studies, it was revealed that inflammatory cytokines, such as interleukin-1β (IL-1β), prostaglandin-E2 (PGE2), and tumor necrosis factor-γ (TNF-γ) were strongly related to the etiology of LBP and the expressions of inflammatory cytokines aforementioned were increased in the degenerated discs [9,11,12]. However, the effect of tensile stress acting on the expression of the inflammatory cytokines has not yet been well studied. Besides, since the inflammatory components are related to pain in intervertebral disc disease, investigating how the tensile stress on the disc affects the production of inflammatory cytokines will shed light on the mechanism of disc degeneration and LBP.

Although the tensile stress on disc cells have been studied *in vitro*, some cytokines including TNF-α [13], interleukin-1(IL-1) [14], nitric oxide (NO) [15], interferon (IFN)-γ, and interleukin-4 (IL-4) [16] have been reported to promote the deterioration of intervertebral disc degeneration. Compared to *in vivo* environments, models *in vitro* were inadequate for imitating the original biomechanical and biologic conditions. Besides, nucleus pulposus (NP) and anulus fibrosus (AF) structures of the intervertebral disc are an entirety, and the changes observed from either part by the tensile stress could not represent its real structural and biomechanical properties. Thus, it is critical to measure the impact of tensile force on inflammatory response in an overall view.

Therefore, the aim of this study is to evaluate the role of tensile stress in the local expression of inflammatory cytokine *in vivo*. The mRNA expressions of IL-17, a typical cytokine participating in the progression of the autoimmune reaction, and inducible NO synthase (iNOS) which regulates the IL-1β production were investigated. IL-1β and IL-17 production by NP and AF in response to tensile stress are discussed in this article.

## Material and methods

### Animals

Our research on animals followed internationally recognized guidelines. The Animal Ethics Committee of Tianjin Hospital approved the study (reference number 2014–009).

Sixty-four female Sprague–Dawley rats (~250 g in weight and 3–3.5 months old) were randomly divided into four groups (*n* = 16): sham, tail-suspended (TS), tail-suspended with needle puncture (TSNP), and single-needle puncture (SNP) groups. The experimental duration was totally 4 weeks. At the end of fourth week, rats were sacrificed and their spines were removed. In each rat, the tail discs between Co7/Co8 and Co8/Co9 (AF and NP together) were carefully removed and saved in liquid nitrogen for enzyme-linked immunosorbent assay (ELISA) and quantitative real-time reverse transcription-polymerase chain reaction (RT-qPCR) analysis.

### Tail suspension

**Figure 1:**
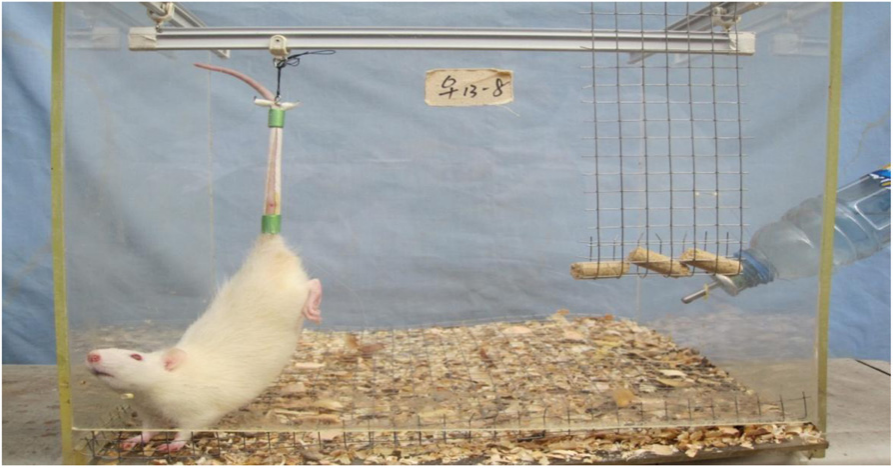
**The rat’s hind limbs are off the cage floor.** The rat could move to every corner of the cage.

Tail suspension was performed using the W.C. Hutton method [17] with some modifications. The rats were placed in modified individual mesh-bottom plastic cages with tail suspension. Each rat was suspended with the tape which was attached to the tail beginning at the base of the tail just above the hair line. The traction tape was gently pressed to the tail so that it sticked along the tail’s surface. The traction tape is narrow enough (~12 in. long and ~0.25 in. wide) so that the tape on one side of the tail does not come into contact with the tape on the other side of the tail. Traction tape was secured to the tail with two strips of filament tape (~0.25 in. wide × 1.5 in. long). One strip of filament tape is placed around the base of the tail over the ends of the traction tape, and a second strip is applied in middle of the tape. The filament tape should be neither too tight to allow normal blood circulation nor too loose to allow the tail to be peeled away. The angle of the body of the rat should be less than 40° head-down. The tape attached to the tail was connected to a pulley linked to a metal arm attached to the center of the top of the cage. Rats suspended in this way had a 360° rotational mobility with full access to the corners of the cage and to food and water (Figure 1). This model could allow us to observe the tensile force being applied on the coccygeal vertebra disc. According to William C. Hutton’s calculation formula, the tensile force value ranged from about 150 g (when the rat was in still) to 450 g (when the rat intended to move forward). Assuming that the rat moved about for a third of the day, and was still (or sleeping) for the other two-thirds, then the average tensile stress in 24 h was 2.45 Newton (N) (2/3 × 150 + 1/3 × 450 = 250 g =2.45 N) [17].

### Tail needle puncture

The coccygeal vertebral disc degeneration and leakage of NP were done by needle puncture according to Bin Han process [18] with some modifications. The needle penetrated into the center of the disc until it reached the opposite side of the tail, and then, needle was rotated 360° and held for 30 s before extraction. This protocol been reported to affect both disc height index and histological score of disc degeneration significantly [18]. The segments of tail puncture were on the disc between the Co7/Co8 and Co8/Co9. The size of needle is 18-g, which has been reported to make the best effect on induction of the disc degeneration [19]. In order to avoid infection, each needle was discarded after single use. The procedure was performed under radiography to ensure that the needle was inserted into the correct place.

### Histologic analysis

At sacrifice, the coccygeal vertebra were removed from the rats, and the discs together with intact adjacent vertebral body bone were fixed in 10% neutral buffered formalin for 1 week, decalcified in ethylenediaminetetraacetic acid and processed for paraffin sectioning. Blocks of tissue were embedded in paraffin and cut into sagittal sections (5 μm in thickness) using a microtome. The sections were stained with hematoxylin and eosin (H&E).

### ELISA

The samples extracted from coccygeal vertebral disc were measured on a jeweler’s scale and then homogenized in 300 μl normal saline containing 0.1% Triton X-100 for 15 min. The homogenates were centrifuged at 15,000 rpm for 10 min. Supernatants were collected by a micropipettes, and ELISA was performed using IL-17 and IL-1β ELISA kit (R&D, Minneapolis, MN, USA) according to the manufacturer’s instructions.

### Extraction of total RNA and reverse transcription to cDNA

After harvesting the discs from rats in each group, total RNA was extracted using UNIQ-10 column (Sangon biotech Co. Ltd., Shanghai, China) according to the manufacturer’s instructions. Reverse transcription was performed using ImProm II Reverse Transcription System (Promega Corporation, Madison, WI, USA).

### RT-qPCR with SYBR green

RT-qPCR was performed using a Prism 7300 (Applied Biosystems Inc, USA) and the SYBR Green Jump Start Taq ReadyMix (Sigma-Aldrich, USA) according to the manufacturer’s specifications. The PCR reaction volume was 20 μL containing 1.5 μL of diluted cDNA and 0.2 μM of each primer. Primers were designed by OligoPerfect Designer (Invitrogen, Valencia, CA, USA) and produced by Nanjing Jin Stewart Biological Technology Co., Ltd, China (see Table 1). The following thermocycling conditions were used: initial polymerase activation step for 2 min at 94°C, followed by 40 cycles of 15 s at 94°C for template denaturation, 1 min at 60°C for annealing, and 1 min at 72°C for extension and fluorescence measurement. All samples were amplified in triplicates and the mean was used for RT-qPCR analysis. Amplification data were analyzed using FlexStation 3 (Molecular Devices, USA). The expressions of the IL-17 and iNOS genes were normalized to that of the endogenous control (GAPDH). Relative levels of target mRNA expression were calculated using the 2^-ΔΔC^T method.

**Table 1:** **Primer sequences used for qRT-PCR**

**Gene name**	**Primer**	**Sequence (5′-3′)**
IL-17	Primer F	TCTTGCCATCTCCATCTTCC
	Primer R	GGGCTTTACTCGAGACACCA
iNOS	Primer F	GCAGCAGCGACTCCATGACT
	Primer R	TCCAGGAGGACATGCAGCAC
GAPDH	Primer F	TGACAACTTTGGCATCGTGG
	Primer R	GGGCCATCCACAGTCTTCTG

### Statistical analysis

One-way analysis of variance was applied to evaluate differences in mean values among various groups. This was followed by pairwise comparisons using a Scheffe correction to compare each condition (TS, TSNP, and SNP) with sham. Student’s *t* test was performed to compare TSNP with SNP specially. All the statistical analyses were conducted using statistical software (SPSS v.16; SPSS Inc., Chicago, IL, USA). A *P* value less than 0.05 was considered significant.

## Results

The rats adapted the tail suspension quite well. All of them were fracture free. The previously experiment had verified the methodology for hanging the rat. The body weights of the rats were stable more than 4 weeks. A constant tensile stress was applied on the coccygeal vertebral disc during the experiment period.

### Histology

**Figure 2:**
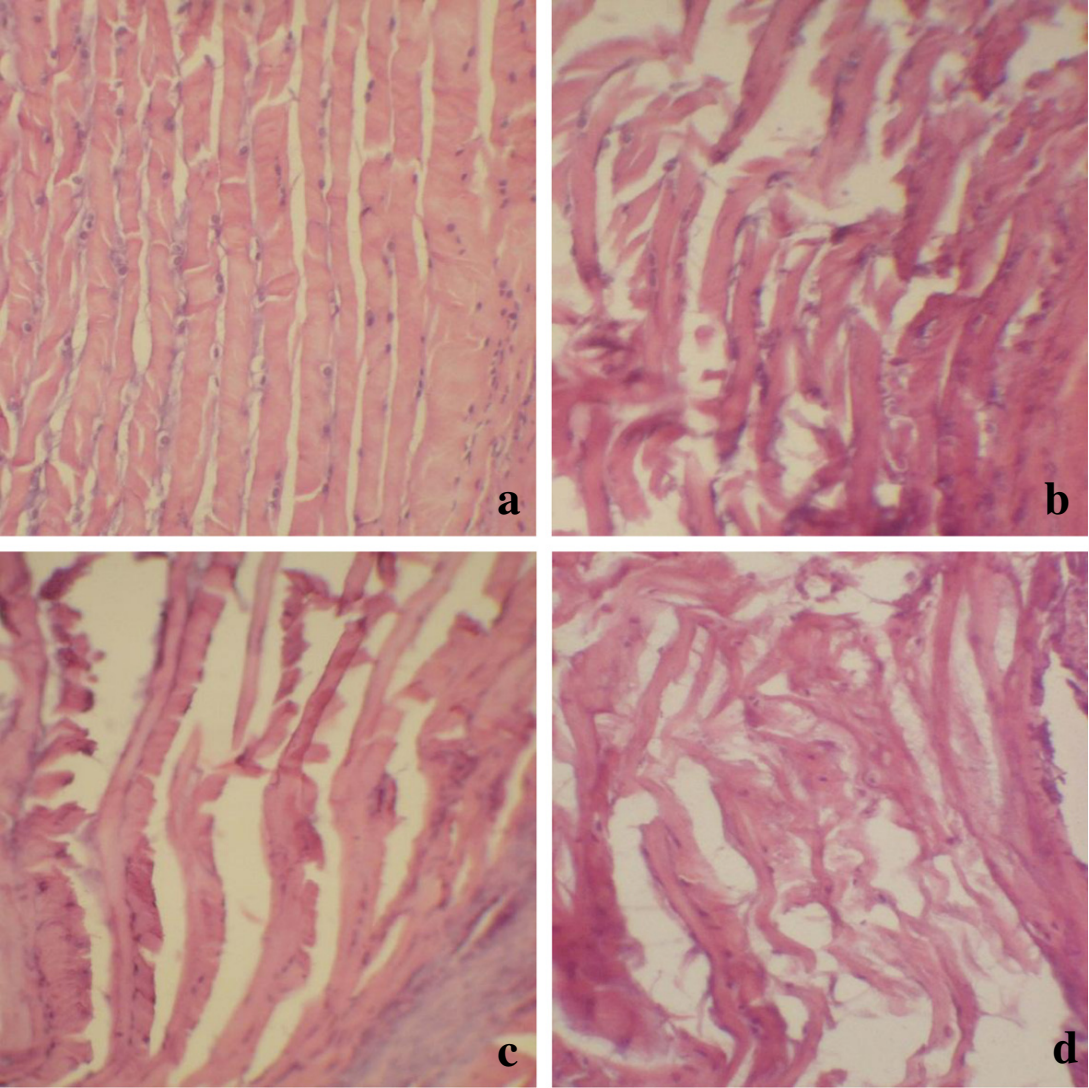
**Representative histologic sections (H&E) of AF in each group. (a)** Sham group, the arrangement of collagenous fiber in AF was quite evenly. **(b)** TS group, collagenous fiber in AF was broken down and malaligned. **(c)** TSNP group, compared to the TS group, the arrangement of AF in TSNP was more turbulent and the fiber spacing was increased and sparsed. **(d)** SNP group, collagenous fiber in AF was mixed and lost the original direction (HE stains × 150).

Figure 2 shows representative histologic sections (H&E) of AF in each group. The AF of sham group was well organized with its lamellar sheets of collagen, the arrangement was quite evenly. In the TS group, collagenous fiber in AF was broken down and malaligned. Compared to the TS group, the arrangement of AF in TSNP was more turbulent and the fiber spacing was increased and sparsed. In the SNP group, collagenous fiber in AF was mixed; cracks and fissures were observed too.

**Figure 3:**
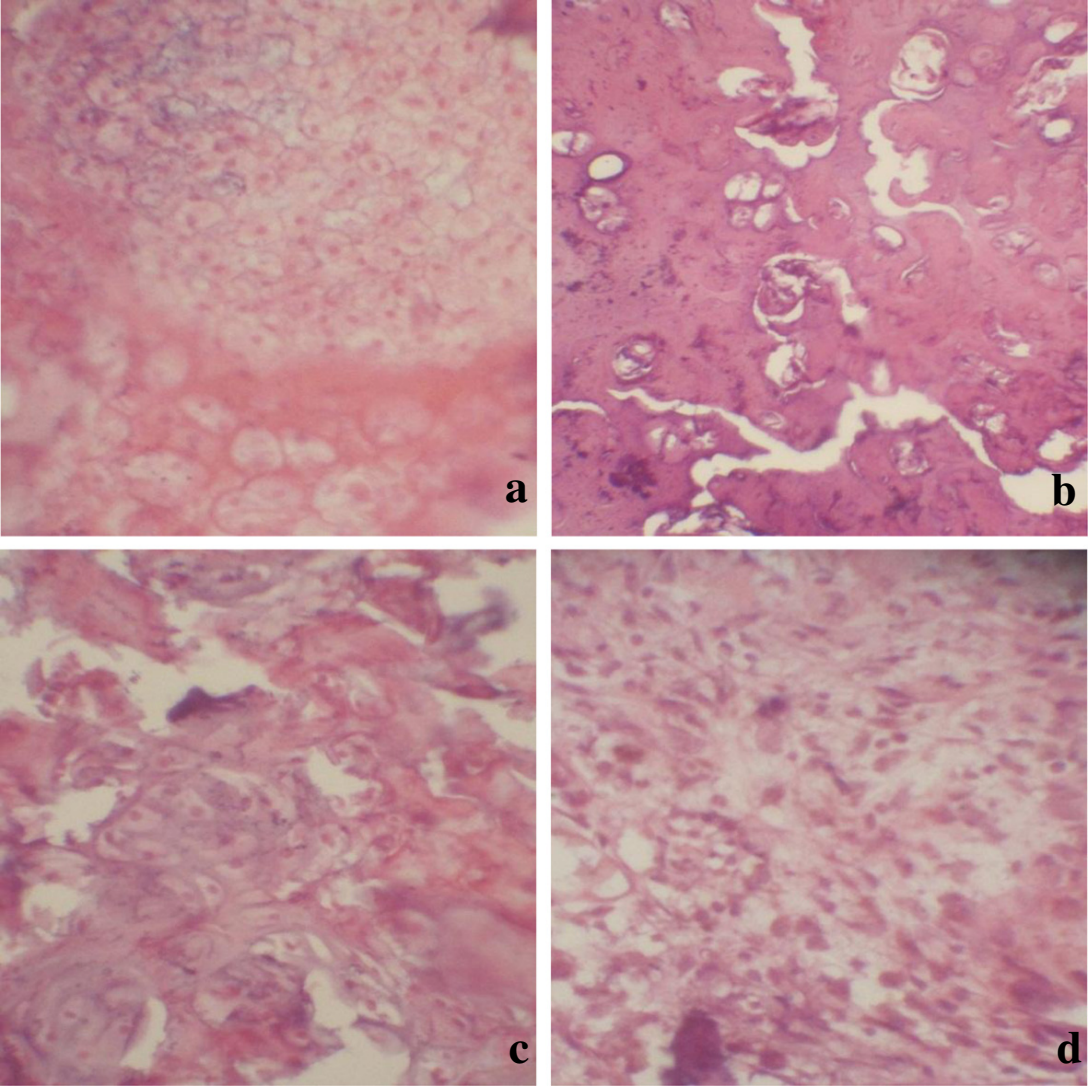
**Histologic sections of NP in sham, TS, SNP, and TSNP groups. (a)** Sham group, the distribution of NP cells were evenly, the size of NP cells were nearly same. **(b)** TS group, the number of the NP decreased with the nuclear cells clustered. **(c)** TSNP group, the figure of NP cells was still, but the size decreased. **(d)** SNP group, the NP cells had smaller size and were scattered and lost the normal structure (HE stains × 150).

Figure 3 indicates the histologic sections of NP in sham, TS, SNP, and TSNP groups after 4 weeks. In the sham group, the distribution of NP cells was even, and the sizes of chondrocyte-like cells were nearly same. In the TS group, the number of the NP decreased while the nuclei of cells clustered. In the TSNP group, the NP exhibited decrease in cell size, but the shape was still fair. In the SNP group, the NP cells had smaller size and were scattered and lost the normal structure.

### Assay of IL-1β and IL-17 productions

ELISA was used to measure immunoreactivity of IL-1β and IL-17 from each group. The average expression in each group is shown in Table 2. In the sham and TS groups, the ELISA failed to detect any specific peptides. In contrast, the average expression of IL-1β and IL-17 in TSNP and SNP groups were significantly upregulated at the fourth week, comparing to the sham and TS groups (*P* < 0.05). A significant lower expression of IL-1β and IL-17 in the TSNP group was observed compared to that in the SNP group (*P* < 0.05).

**Table 2:** **Average results of ELISA for IL-1β and IL-17 for the four groups of rats (pg/g)**

**Group**	**Number of samples**	**IL-1β**	**IL-17**
Sham	16	Negative	Negative
TS	16	Negative	Negative
TSNP	16	8.74 ± 4.10*	12.85 ± 4.09*
SNP	16	17.36 ± 5.93*	34.07 ± 7.47*

The levels of immunodetectable IL-1β and IL-17 in the coccygeal vertebral discs were determined by ELISA as described in the ‘Methods’ section. The values were shown as mean ± SD of data from each experiment. Significant differences were observed.

*P < 0.05.

### IL-17 and iNOS gene expressions in each condition (TS, TSNP, and SNP) compared to sham

**Figure 4:**
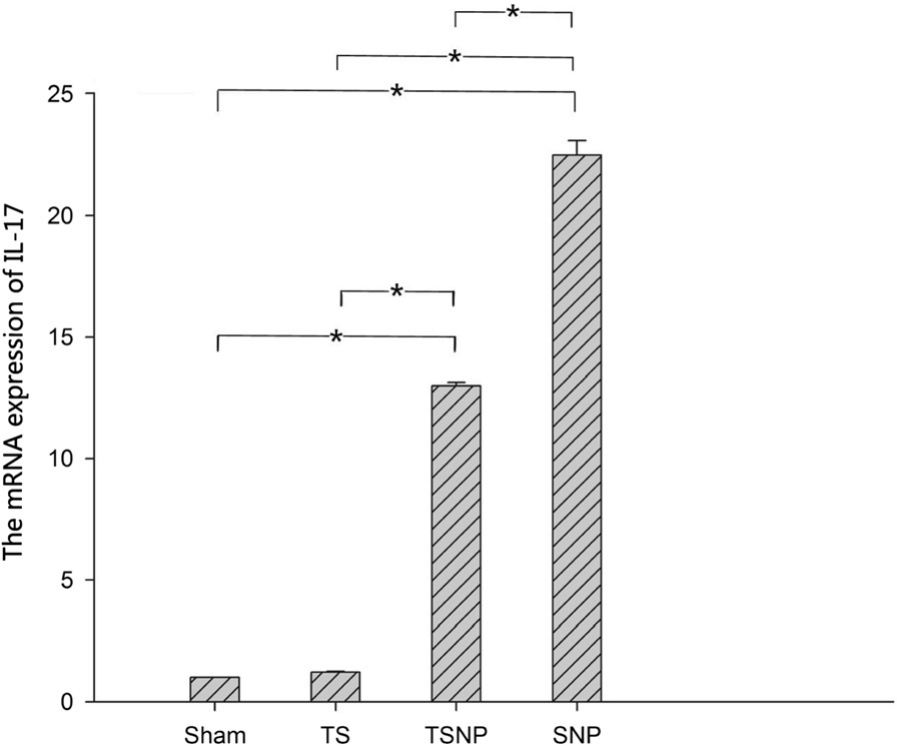
**Both expressions of mRNA in TSNP and SNP groups tend to rise significantly compared to the sham group (** ***P*** **< 0.05).** On the contrary, the level of TS group did not show the significant differences (*P* > 0.05). Compared with the SNP group, the expression of IL-17 in TSNP group decreased significantly (*P* < 0.05).

**Figure 5:**
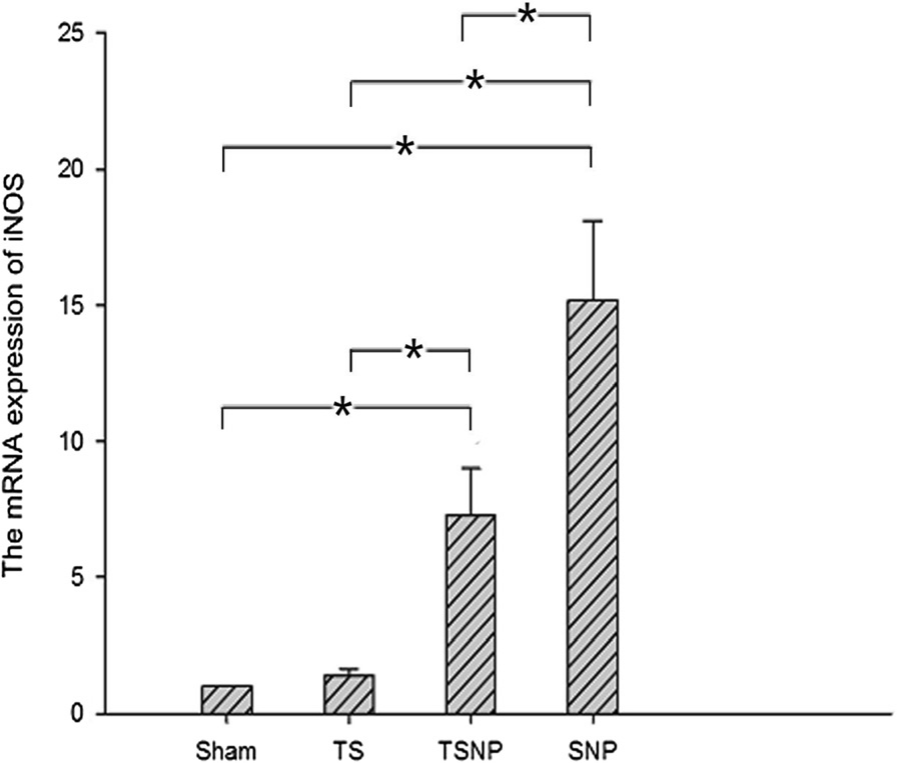
**Both expressions of mRNA in TSNP and SNP groups tend to rise significantly compared to the sham group (** ***P*** **< 0.05).** On the contrary, the level of TS group did not show the significant differences (*P* > 0.05). Compared with the SNP group, the expression of iNOS in TSNP group decreased significantly (*P* < 0.05).

In the disc, the IL-17 and iNOS mRNA levels were significantly higher in both TSNP and SNP groups than in the corresponding sham group (*P* < 0.05, Figures 4 and 5). More than 13-fold and 7-fold increases in the mRNA expressions of IL-17 and iNOS, respectively, were observed in the TSNP group, compared to the sham groups. More than 22-fold and 15-fold increases in the mRNA expressions of IL-17 and iNOS, respectively, were observed in SNP group, compared to the sham groups. Sole application of tensile stress brought by the tail suspension slightly enhanced the expressions of both IL-17 and iNOS. But the differences were not significant comparing to the control groups (*P* > 0.05).

Compared with the SNP group, the tensile stress in the TSNP group represses the mRNA expression of IL-17 and iNOS. Levels of IL-17 and iNOS between two groups were significantly different. (*P* < 0.05, Figures 4 and 5).

## Discussion

A previous study reported the beneficial effects of motion on the spine in patients with low-back pain from the clinical perspective [20], and some studies have also verified the roles of tensile stress on the disc *in vitro* [11,21]. These clinical and experimental outcomes provided strong supports to our model system which was focusing on the changes of inflammatory cytokines after the application of relatively low magnitude of tensile stress through a novel *in vivo* animal model. To the best of our knowledge, this is the first report evaluating the impact of tensile stress on the expression of inflammatory factor in the rat coccygeal vertebral disc *in vivo*.

From the results, the mRNA expressions of IL-17 and iNOS in the TS groups showed a mild rise but insignificantly different compared with that in the sham group (*P* > 0.05). The TSNP and SNP groups, however, both show significant increases in expression when compared with the control and TS groups (*P* < 0.05). It was found that needle puncture to rat coccygeal vertebrae disc could induce the soaring of inflammatory factors by leakage from NP and the breakage annular, which was similar to the disease of human intervertebral disc herniation. Anular penetration has been used to induce simulated degeneration in rodent models [19,22], can destroy the integrity of the intervertebral disc, and further cause the leak of NP. The NP is a sort of immune-privilege tissue with vascular isolation from birth and would be recognized as a foreign antigen that induces an autoimmune response producing inflammation once herniation occurred [23,24]. The tail suspension model provides a stable and better-controlled tensile stress on the coccygeal vertebral disc.

Recent studies have showed the effect of T helper 17 (Th17) lymphocytes regarding the inflammatory procession and autoimmune disease, such as rheumatoid arthritis, Crohn’s disease, etc. [25,26]. Th17 lymphocytes primarily produce IL-17, a kind of pro-inflammatory cytokine, which is mediated by the production of IL-23. IL-1β is also a major mediator of inflammation and can be found in the synovial fluids of inflamed joints [27]. IL-1β exerts its biological function via production of NO [28], and the synthesis of NO is regulated by expression of iNOS [28,29]. Given the potential capability of IL-17 and iNOS to upregulate PGE2 and IL-1β, which causes pain [30] or raise the sensibility to algesia [31], IL-17 and iNOS may be the candidates for exerting an influence on LBP.

This study also showed that comparing to the SNP group, the mRNA expressions of IL-17 and iNOS, as well as the secretion of IL-17 and IL-1β in the TSNP group, were significantly repressed (*P* < 0.05). These results indicated that tensile stress could efficaciously restrain the production of IL-17 and IL-1β in the disc cells. Although the capacity of inflammation induced by NP has been evaluated by several studies [32-34], how the tensile stress impacts the inflammatory properties of disc was still unknown. The results from our study suggested that the tensile stress played a positive role to relieving the process of inflammation and pain through downregulation of IL-17 and iNOS. This finding is consistent with the former discovery of Holm S et al. [35], who evaluated the role of motion on the intervertebral disc and found a beneficial effect through increasing diffusion, accelerating waste exchange, and resulting in enhanced nutrition. Sowa G et al. [11] testified the relationship between low levels of tensile stress and the degree of inflammation *in vitro*. Their findings revealed that the volume of inflammatory cytokines such as matrix metalloprotease-13 (MMP-13) and collagen II decrease significantly in response to tensile stress. Mechanical stress may act the anti-inflammatory role in the environment of inflammation. Considering these experimental evidences and the results of the current study together, there was no surprise that motion therapy, such as traction, is able to lighten the symptom of LBP by controlling the expression of inflammatory cytokines.

We selected the tail-suspension method to exert tensile stress on the disc because it not only provided a constant and suitable stress [17] but also was easy to be adapted by the rat [36]. An appropriate range of tensile stress applied on the disc was vital to impact the inflammatory response. In our experiment, the mechanical stress applied on the tail through suspension device was approximate 2.45 N. Such relatively low magnitude of tensile stress was commonly used in clinical practice, showing a positive effect in reducing not only the amount of IL-17 and IL-1β but also mRNA expressions of IL-17 and iNOS. This result was coincident with a few previous literatures: Lai, A. et al. [37] discovered that low magnitude (1.4 N) could hold the proper figures of the intervertebral disc, whereas traction of relatively high magnitude (4.2 N) showed adverse effects to the disc. Moreover, tensile stress of 6% elongation applied to the cultured rat coccygeal disc could decrease inducible inflammatory gene expression [11], but tensile stress of 20% elongation would enhance some key enzymes in the process of inflammatory mediator synthesis [9]. Those results inspired us that the level of tensile stress ought to be taken into account when we are treating the LBP patients with traction.

Regarding the methodology of the current study, this animal model provides us with a more adaptable and stable experimental platform to evaluate the effects of tensile stress on inflammation in various ways. Due to the significant structure differences between the rat-tail discs and human intervertebral discs, the results of this study could not directly reflect the authentic changes of inflammatory cytokines under tensile stress in human though this experiment was applied *in vivo*. Besides, to explore the further mechanism of how tensile stress influences the inflammatory cytokines in the vertebral disc, various amount of tensile stress, as well as the duration and/or a dose-dependence of inflammatory cytokines in respond to mechanical strain, should be considered. Finally, the tensile stress applied on the disc by tail suspension was not constant; following the movement of rats, the value of tensile stress could regularly change. What we actually measured was the mean value during the whole day. If there is a new apparatus that can control the tensile stress precisely with good adaptivity to the rat, it would be better to illuminate the accurate effect of tensile stress on inflammatory response and LBP.

## Conclusions

This study investigated the effect of relatively low magnitude of tensile stress on the changes of IL-1β and IL-17. It was demonstrated that mechanical strain with relatively low magnitude had a significant positive effect in the spinal disease by restraining the degree of inflammation. Although tensile stress was not able to reverse the process of disease in intervertebral disc, the results suggested that the tensile stress in low magnitude played a positive role in relieving the symptom of LBP. Future studies of the effects of tensile strain on discs could provide a potential new therapeutics for LBP.

## References

[1] Revel M (2000). Does traction still have a role in nonspecific low back disorders?. Joint Bone Spine..

[2] Long A, Donelson R, Fung T (2004). Does it matter which exercise? A randomized control trial of exercise for low back pain. Spine (Phila Pa 1976).

[3] Borman P, Keskin D, Bodur H (2003). The efficacy of lumbar traction in the management of patients with low back pain. Rheumatol Int..

[4] Beurskens AJ, De Vet HC, Koke AJ, Regtop W, van der Heijden GJ, Lindeman E (1997). Efficacy of traction for nonspecific low back pain. 12-week and 6-month results of a randomized clinical trial. Spine (Phila Pa 1976).

[5] Elliott DM, Sarver JJ (2004). Young investigator award winner: validation of the mouse and rat disc as mechanical models of the human lumbar disc. Spine (Phila Pa 1976).

[6] Rannou F, Richette P, Benallaoua M, Francois M, Genries V, Korwin-Zmijowska C (2003). Cyclic tensile stretch modulates proteoglycan production by intervertebral disc annulus fibrosus cells through production of nitrite oxide. J Cell Biochem..

[7] Hirukawa K, Miyazawa K, Maeda H, Kameyama Y, Goto S, Togari A (2005). Effect of tensile force on the expression of IGF-I and IGF-I receptor in the organ-cultured rat cranial suture. Arch Oral Biol..

[8] Yang G, Crawford RC, Wang JH (2004). Proliferation and collagen production of human patellar tendon fibroblasts in response to cyclic uniaxial stretching in serum-free conditions. J Biomech..

[9] Miyamoto H, Doita M, Nishida K, Yamamoto T, Sumi M, Kurosaka M (2006). Effects of cyclic mechanical stress on the production of inflammatory agents by nucleus pulposus and anulus fibrosus derived cells *in vitro*. Spine (Phila Pa 1976).

[10] Lotz JC, Ulrich JA (2006). Innervation, inflammation, and hypermobility may characterize pathologic disc degeneration: review of animal model data. J Bone Joint Surg Am..

[11] Sowa G, Agarwal S (2008). Cyclic tensile stress exerts a protective effect on intervertebral disc cells. Am J Phys Med Rehabil..

[12] Igarashi T, Kikuchi S, Shubayev V, Myers RR (2000). 2000 Volvo Award winner in basic science studies: Exogenous tumor necrosis factor-alpha mimics nucleus pulposus-induced neuropathology. Molecular, histologic, and behavioral comparisons in rats. Spine (Phila Pa 1976).

[13] Weiler C, Nerlich AG, Bachmeier BE, Boos N (2005). Expression and distribution of tumor necrosis factor alpha in human lumbar intervertebral discs: a study in surgical specimen and autopsy controls. Spine (Phila Pa 1976).

[14] Le Maitre CL, Hoyland JA, Freemont AJ (2007). Catabolic cytokine expression in degenerate and herniated human intervertebral discs: IL-1beta and TNFalpha expression profile. Arthritis Res Ther..

[15] Katsuno R, Hasegawa T, Iwashina T, Sakai D, Mikawa Y, Mochida J (2008). Age-related effects of cocultured rat nucleus pulposus cells and macrophages on nitric oxide production and cytokine imbalance. Spine (Phila Pa 1976).

[16] Geiss A, Larsson K, Junevik K, Rydevik B, Olmarker K (2009). Autologous nucleus pulposus primes T cells to develop into interleukin-4-producing effector cells: an experimental study on the autoimmune properties of nucleus pulposus. J Orthop Res..

[17] Hutton WC, Yoon ST, Elmer WA, Li J, Murakami H, Minamide A (2002). Effect of tail suspension (or simulated weightlessness) on the lumbar intervertebral disc: study of proteoglycans and collagen. Spine (Phila Pa 1976).

[18] Han B, Zhu K, Li FC, Xiao YX, Feng J, Shi ZL (2008). A simple disc degeneration model induced by percutaneous needle puncture in the rat tail. Spine (Phila Pa 1976).

[19] Hsieh AH, Hwang D, Ryan DA, Freeman AK, Kim H (2009). Degenerative anular changes induced by puncture are associated with insufficiency of disc biomechanical function. Spine (Phila Pa 1976).

[20] Daenen L, Varkey E, Kellmann M, Nijs J (2015). Exercise, not to exercise, or how to exercise in patients with chronic pain? Applying science to practice. Clin J Pain..

[21] Terahata N, Ishihara H, Ohshima H, Hirano N, Tsuji H (1994). Effects of axial traction stress on solute transport and proteoglycan synthesis in the porcine intervertebral disc *in vitro*. Eur Spine J..

[22] Korecki CL, Costi JJ, Iatridis JC (2008). Needle puncture injury affects intervertebral disc mechanics and biology in an organ culture model. Spine (Phila Pa 1976).

[23] Gertzbein SD, Tile M, Gross A, Falk R (1975). Autoimmunity in degenerative disc disease of the lumbar spine. Orthop Clin North Am..

[24] Bobechko WP, Hirsch C (1965). Auto-immune response to nucleus pulposus in the rabbit. J Bone Joint Surg (Br)..

[25] Weaver CT, Hatton RD, Mangan PR, Harrington LE (2007). IL-17 family cytokines and the expanding diversity of effector T cell lineages. Annu Rev Immunol..

[26] Moseley TA, Haudenschild DR, Rose L, Reddi AH (2003). Interleukin-17 family and IL-17 receptors. Cytokine Growth Factor Rev..

[27] Firestein GS, Boyle DL, Yu C, Paine MM, Whisenand TD, Zvaifler NJ (1994). Synovial interleukin-1 receptor antagonist and interleukin-1 balance in rheumatoid arthritis. Arthritis Rheum..

[28] Stichtenoth DO, Frolich JC (1998). Nitric oxide and inflammatory joint diseases. Br J Rheumatol..

[29] Hauselmann HJ, Stefanovic-Racic M, Michel BA, Evans CH (1998). Differences in nitric oxide production by superficial and deep human articular chondrocytes: implications for proteoglycan turnover in inflammatory joint diseases. J Immunol..

[30] Taiwo YO, Levine JD (1990). Effects of cyclooxygenase products of arachidonic acid metabolism on cutaneous nociceptive threshold in the rat. Brain Res..

[31] Whelan CJ, Head SA, Poll CT, Coleman RA (1991). Prostaglandin (PG) modulation of bradykinin-induced hyperalgesia and oedema in the guinea-pig paw–effects of PGD2, PGE2 and PGI2. Agents Actions Suppl..

[32] McCarron RF, Wimpee MW, Hudkins PG, Laros GS (1987). The inflammatory effect of nucleus pulposus. A possible element in the pathogenesis of low-back pain. Spine (Phila Pa 1976).

[33] Olmarker K, Rydevik B, Nordborg C (1993). Autologous nucleus pulposus induces neurophysiologic and histologic changes in porcine cauda equina nerve roots. Spine (Phila Pa 1976).

[34] Geiss A, Larsson K, Rydevik B, Takahashi I, Olmarker K (2007). Autoimmune properties of nucleus pulposus: an experimental study in pigs. Spine (Phila Pa 1976).

[35] Holm S, Nachemson A (1983). Variations in the nutrition of the canine intervertebral disc induced by motion. Spine (Phila Pa 1976).

[36] Morey-Holton ER, Globus RK (2002). Hindlimb unloading rodent model: technical aspects. J Appl Physiol..

[37] Lai A, Chow DH (2010). Effects of traction on structural properties of degenerated disc using an in vivo rat-tail model. Spine (Phila Pa 1976).

